# Recruiting Medical, Dental, and Biomedical Students as First Responders in the Immediate Aftermath of the COVID-19 Pandemic: Prospective Follow-Up Study

**DOI:** 10.2196/63018

**Published:** 2025-04-24

**Authors:** Nicolas Schnetzler, Victor Taramarcaz, Tara Herren, Eric Golay, Simon Regard, François Mach, Amanta Nasution, Robert Larribau, Melanie Suppan, Eduardo Schiffer, Laurent Suppan

**Affiliations:** 1 Department of Anaesthesiology, Pharmacology, Intensive Care and Emergency Medicine, Faculty of Medicine, University of Geneva, Geneva, Switzerland; 2 Division of Emergency Medicine, Department of Acute Care Medicine, Geneva University Hospitals, Rue Gabrielle-Perret-Gentil 2, Geneva, 1211, Switzerland, 41 223723311; 3 Cantonal Physician Division, Cantonal Health Office, State of Geneva, Geneva, Switzerland; 4 Cardiology Department, University of Geneva Hospitals and Faculty of Medicine, Geneva, Switzerland; 5 Division of Anaesthesiology, Department of Acute Care Medicine, Geneva University Hospitals, Geneva, Switzerland; 6 Unit of Development and Research in Medical Education (UDREM), Faculty of Medicine, Geneva, Switzerland

**Keywords:** basic life support, out-of-hospital cardiac arrest, cardiopulmonary resuscitation, e-learning, blended learning, first responder, undergraduate medical education, student motivation, motivational strategies, medical student, COVID-19, pandemic, life support, survival prognosis, biomedical students, dental students, motivational interventions

## Abstract

**Background:**

Basic life support improves survival prognosis after out-of-hospital cardiac arrest, but is too rarely provided before the arrival of professional rescue services. First responder networks have been developed in many regions of the world to decrease the delay between collapse and initiation of resuscitation maneuvers. Their efficiency depends on the number of first responders available and many networks lack potential rescuers. Medical, dental, and biomedical students represent an almost untapped source of potential first responders, and a first study, carried out during the COVID-19 pandemic, led to the recruitment of many of these future professionals even though many restrictions were still in effect.

**Objective:**

The objective of this study was to determine the impact of an enhanced strategy on the recruitment of medical, dental, and biomedical students as first responders in the immediate aftermath of the COVID-19 pandemic.

**Methods:**

This was a prospective follow-up study, conducted between November 2021 and March 2022 at the University of Geneva Faculty of Medicine, Geneva, Switzerland. A web-based study platform was used to manage consent, registrations, and certificates. A first motivational intervention was held early in the academic year and targeted all first-year medical, dental, and biomedical students. Participants first answered a questionnaire designed to assess their initial basic life support knowledge before following an e-learning module. Those who completed the module were able to register for a face-to-face training session held by senior medical students. A course certificate was awarded to those who completed these sessions, enabling them to register as first responders on the Save a Life first responder network. Since the number of students who had enlisted as first responders 2 months after the motivational intervention was markedly lower than expected, a second, unplanned motivational intervention was held in an attempt to recruit more students.

**Results:**

Out of a total of 674 first-year students, 19 (2.5%) students had registered as first responders after the first motivational intervention. This was significantly less than the proportion achieved through the initial study (48/529, 9.1%; *P*<.001). The second motivational intervention led to the enrollment of 7 more students (26/674, 3.9%), a figure still significantly lower than that of the original study (*P*<.001). At the end of the study, 76 (11.3%) students had been awarded a certificate of competence.

**Conclusions:**

Contrary to expectations, an earlier presentation during the academic year outside the COVID restriction period did not increase the recruitment of medical, dental, and biomedical students as first responders in the immediate aftermath of the COVID-19 pandemic. The reasons underlying this drop in motivation should be explored to enable the design of focused motivational interventions.

## Introduction

### Background

Basic life support (BLS) improves survival prognosis after out-of-hospital cardiac arrest (OHCA) but is too rarely provided before the arrival of professional rescue services [1-6]. Without BLS, the probability of survival decreases by 10% for each minute that passes [7]. Thus, professional rescue is of limited worth if BLS has not been provided either by bystanders or by first responders [8-10]. Indeed, several studies have demonstrated that initiation of BLS maneuvers by nonprofessionals improves survival and neurological outcomes [1,11,12].

Increasing global awareness regarding the importance of quickly initiating BLS maneuvers after OHCA will take time, and barriers to action often prevent bystanders from initiating cardiopulmonary resuscitation [1,13-15]. To overcome this limitation, first responder systems have been developed in many regions of the world. These systems rely on BLS-certified professional or nonprofessional rescuers who accept a call to respond to OHCA alarms if they happen to be nearby.

In Geneva, Switzerland, the Save a Life project was initiated in October 2019 by the Swiss Emergency Responder Association, with the objective of developing a regional network of first responders [7]. When an OHCA is identified by the emergency medical call center, an alert is displayed on the Save a Life first responder app. If a first responder is near enough and agrees to intervene, the position of the nearest automatic external defibrillator (AED) is displayed along with the exact location of the intervention. The main limitations of this system are the limited number of first responders, their availability, and their geographical distribution.

To improve the number of first responders, Taramarcaz et al [16] designed a process to recruit first-year medical students. Their study took place while the COVID-19 pandemic was still ongoing, and many restrictions were still in effect. In addition, the motivational intervention designed to catch the students’ interest was held online rather than in an auditorium and took place rather late after the beginning of the academic year and close to a critical exam session. Thus, the authors hypothesized that an intervention taking place earlier in the academic year, and without the constraints imposed by the COVID-19 pandemic, could lead to higher participation rates and the recruitment of a higher proportion of medical students as first responders [16].

### Objective

The objective of this study was to determine the impact of the modifications proposed by Taramarcaz et al [16] on first responder recruitment.

## Methods

### Study Design

This prospective follow-up study was conducted between November 2021 and March 2022 and followed a structure and sequence similar to that described by Taramarcaz et al [16].

The study platform used for the initial study was reset and reused for this follow-up study. Given the use of a web-based platform, methods and results are reported according to the Checklist for Reporting Results of Internet E-Surveys (CHERRIES) guidelines when appropriate [17]. Data were managed in accordance with the European General Data Protection Regulation [18]. A more detailed description of the tools used can be found in Multimedia Appendix 1.

The learning path was identical to that described in Taramarcaz et al’s [16] study and followed a flipped classroom design: after ensuring that no exclusion criteria were present, the first-year medical, dental, and biomedical students of the University of Geneva Faculty of Medicine (UGFM) answered a questionnaire designed to assess their initial BLS knowledge before following an e-learning module. After completing this module, they were able to register for a face-to-face training session held by senior medical students. The estimated time required to complete the e-learning and practice session was about an hour and a half. This duration was chosen as being long enough for learning and skill retention while avoiding an overt demand on their busy schedule. The participants who completed the entire learning path were awarded a BLS-AED course certificate enabling them to register as first responders on the Save a Life first responder network. The whole process, including the certification, was entirely free of charge, and there was no obligation for students to participate. The only incentive was to obtain a BLS-AED certificate.

### Ethical Considerations

Since the regional ethics committee (Commission cantonale d’éthique de la recherche, Geneva, Switzerland) had already acknowledged that this design did not fall within the scope of the Swiss federal law on research involving human beings (Req-2020‐01143), no further ethical assessment was required or requested.

### Recruitment

A motivational intervention was performed live on November 29, 2021. This intervention was animated by 2 senior medical students and took place at the end of a basic medical science course. The presentation contained a short, humorous introductory video, a description of the project, and an overview of the Save a Life network. The last slide included a QR code and a URL link to the study platform as indicated in the research protocol [19]. On the same day, all potential participants received the same information by email through the class’s mailing list. To promote participation, a second motivational email was sent to the entire class on December 10.

Since participation was markedly lower than expected by the end of December, a second intervention was planned, this time at the start of a course on atherosclerosis given by the head of the cardiology department at Geneva University Hospital. This intervention took place on January 10, 2022, and a final reminder email was sent on January 13, 2022. For the second intervention, different support material was used, and various real-life scenarios were included, showcasing how BLS knowledge could enable them to act in the case of OHCA.

### Enrollment

The QR code and URL provided during the motivational interventions and through the invitation emails redirected the students to an introductory page detailing the study’s objectives and procedures. Those willing to participate were asked to answer 2 questions designed to detect the presence of either of 2 exclusion criteria: being registered as first responder and not being a UGFM student. If neither exclusion criterion was met, the students were redirected to a consent form (Table 1) including a disclaimer about data handling and security. Those who agreed were asked to create an account and to provide minimal personal information (first name, last name, and email address) for contact purposes and to allow for the creation of nominative BLS-AED certificates. The students who refused to participate and those who met either exclusion criteria were also given the possibility to follow the learning path and to receive a certificate allowing them to join a first responder system.

After completing the registration process, participants were asked to fill out a precourse questionnaire designed to gather demographic data and determine their precourse BLS knowledge (Table 2).

**Table 1.** Screening questionnaire and consent form (reused from Taramarcaz et al [16]).

Survey page, field, and question	Type of question
Page 1	
Already filled the questionnaire or exclusion criteria	
Already a first responder	Yes or no
Demographics	
Student at UGFM^a^	Yes or no
If no: current professional status	Open
Consent	
Agree to participate	Yes or no
If no: reasons for refusal	MAQ^b^
If no: access to the e-learning module	Yes or no

a UGFM: University of Geneva Faculty of Medicine.

b MAQ: multiple answer question.

**Table 2.** Precourse questionnaire (reused from Taramarcaz et al [16]).

Survey page, field, and question	Type of question
1: Demographics	
Year of birth	Open (Regex^a^)
Gender	MCQ^b^
Medical, biomedical or dental medicine student	MCQ
Former student or graduate of another health care profession	MCQ
Target Specialty	MCQ
2: General BLS^c^ knowledge	
Ever heard of BLS or ACLS^d^ before	Yes/no
Meaning of AED^e^^,^^f^	Open
Year of the last BLS guidelines update	Open (Regex)
Phone number of the emergency medical communication center^f^	Open
3: Prior BLS experience	
Prior BLS training	MAQ^g^
Wish for additional BLS training	Yes/no
4: Specific BLS knowledge	
Criteria used to recognize OHCA^f^^,^^h^	MAQ
BLS-sequence^f^	Ordering
Artery for pulse assessment^f^	MCQ
Compression depth^f^	MCQ
Compressions: ventilation ratio^f^	MCQ
Compression rate^f^	MCQ
Compression-only CPR^f^^i^	Yes/no
Foreign body airway obstruction ^f^	MCQ
5: Confidence	
Precourse confidence to act in an OHCA situation	Likert scale (1-5)

a A Regex validation rule was used to avoid invalid entries.

b MCQ: multiple choice question (only one answer accepted).

c BLS: basic life support.

d ACLS: advanced cardiovascular life support.

e AED: automatic external defibrillator.

f Items used to calculate the 10-point score (initial BLS knowledge).

g MAQ: multiple answer question (more than one answer accepted).

h OHCA: out-of-hospital cardiac arrest.

i CPR: cardiopulmonary resuscitation.

### E-Learning and Practice Sessions

The interactive e-learning module used in Taramarcaz et al’s [16] study was reused without any changes since it still matched the objectives, respected the Swiss Resuscitation Council’s guidelines, and had not received any negative feedback from the students. This module was designed to last about 30 minutes, but no time limit was set and students were able to resume at will. A screen enabling participants to register for near-peer animated practice sessions was displayed upon completion of this e-learning module.

Practice sessions lasted 1 hour and were limited to 4 participants. A total of 32 sessions (128 slots) were planned between December 6, 2021 and March 11, 2022. The instructor-to-participant ratio (1:4) was kept unchanged to maintain high-quality training even though the COVID-19 restrictions had been lifted. The senior medical students who animated these near-peer-led practice sessions were all certified as BLS-AED instructors according to the Swiss Resuscitation Council’s guidelines. Most of the students who had already participated as instructors in Taramarcaz et al’s [16] study (15/17, 88%) agreed to resume their involvement and 5 new instructors were trained. While all instructors were to ensure that the objectives had been met by using a standardized checklist, they were free to adapt the structure of their training sessions according to the participants’ profiles.

### Final Questionnaire and Certification

An email embedding a link to a postcourse questionnaire was sent to the students who successfully completed the practice sessions (Table 3). Participation in this questionnaire was mandatory to obtain a nominative BLS-AED certificate. These certificates, which had a 1-year validity, enabled participants to enroll as first responders on the Save a Life platform.

**Table 3.** Postcourse questionnaire (reused from Taramarcaz et al [16]).

Survey page, field, and question	Type of question
1: Opinion	
Appreciation	Yes/no
If yes: positive thoughts	MAQ^a^
If no: negative thoughts	MAQ
General comments	Free text
2: Confidence	
Postcourse confidence for OHCA^b^ management	Likert scale (1-5)
Factors contributing to confidence	Likert scale (1-5)
Factors contributing to lack of confidence	Likert scale (1-5)
Other comments on confidence	Free text
3: First responders	
Intention to register as first responder	Yes/no
If yes: contributing factors	Likert scale (1-5)
If no: impeding factors	Likert scale (1-5)
Other factors	Free text
4: Improvement	
Suggestion for improvement	Free text

a MAQ: multiple answer question.

b OHCA: out-of-hospital cardiac arrest.

### Adaptations From the Implementation Study

In line with this study’s objectives, the main changes from the implementation study were that the initial presentation to first-year students and the training sessions were held earlier in the academic year [16], with the hypothesis that this would increase the number of registrations as first-year students would be further away from their final exams. Thus, the project was presented on November 29, 2 months earlier than the original study.

Despite this adaptation, and contrarily to our hypothesis, the number of participants was markedly lower than that in the original study. A second, initially unplanned intervention was therefore carried out in early January 2022, and constitutes the second major adaptation from the original implementation study.

Another difference was that biomedical students were also invited to participate in this study. Finally, the practice sessions were held between December 2021 and March 2022 in this study while they had taken place between January and April 2021 in the implementation study. The number of slots remained unchanged.

### Outcomes

The primary outcome was the proportion of students who had registered as first responders before the second intervention took place, that is, by January 9, 2022. Secondary outcomes were the proportion of students who had registered following the second intervention, the overall proportion of students who had registered as first responders by May 1, 2022, and attrition at each step of the study [20]. The difference in self-reported confidence in performing BLS maneuvers was also assessed.

### Statistical Analysis

Data curation and analysis were carried out using STATA/BE (version 17.0; StataCorp LLC). Descriptive statistics were used to describe the evolution of the number of students at each step of the learning path. Given the sample size, parametric tests were used when appropriate. A *P* value of less than .05 was considered statistically significant.

The chi-square test was used to assess the difference in student recruitment distribution between this study and Taramarcaz et al’s [16]. This was carried out by reusing the original data file, which is freely available online as a Multimedia Appendix 1 of the original study. Since biomedical students had not been invited to participate in the original study, a sensitivity analysis was carried out by excluding them.

Potential differences between students who registered after following the first motivational intervention and those who registered after following the second one were looked for by applying a *t* test on the 10-point BLS score and by comparing attrition at each step. No weighting was used to compute the 10-point BLS knowledge score. A *t* test was performed to look for a difference between this score and interest in following BLS training.

A *t* test was also used to investigate whether there was a statistically significant difference between postcourse confidence and enrollment in the first responder network.

Given the presence of cells with very limited numbers (<5), Fischer tests were applied to analyze the factors influencing self-confidence and the desire to join the Save a Life first responder network.

## Results

The 2021‐2022 academic year included a total of 674 first-year students at UGFM in human and dental medicine and in biomedical sciences. The proportion of students who had registered as first responders after the first motivational intervention was 2.5% (19/674), significantly less than after Taramarcaz et al’s [16] implementation study (48/529, 9.1%; *P*<.001). The second motivational intervention led to the enrollment of 7 more students (26/674, 3.9%). This figure is still significantly lower than that observed in Taramarcaz et al’s study (*P*<.001) [16]. Even after excluding biomedical students from the analysis, the figure remained significantly lower (25/600, 4.2%) than in Taramarcaz et al’s [16] study (*P*=.001).

**Figure 1.**
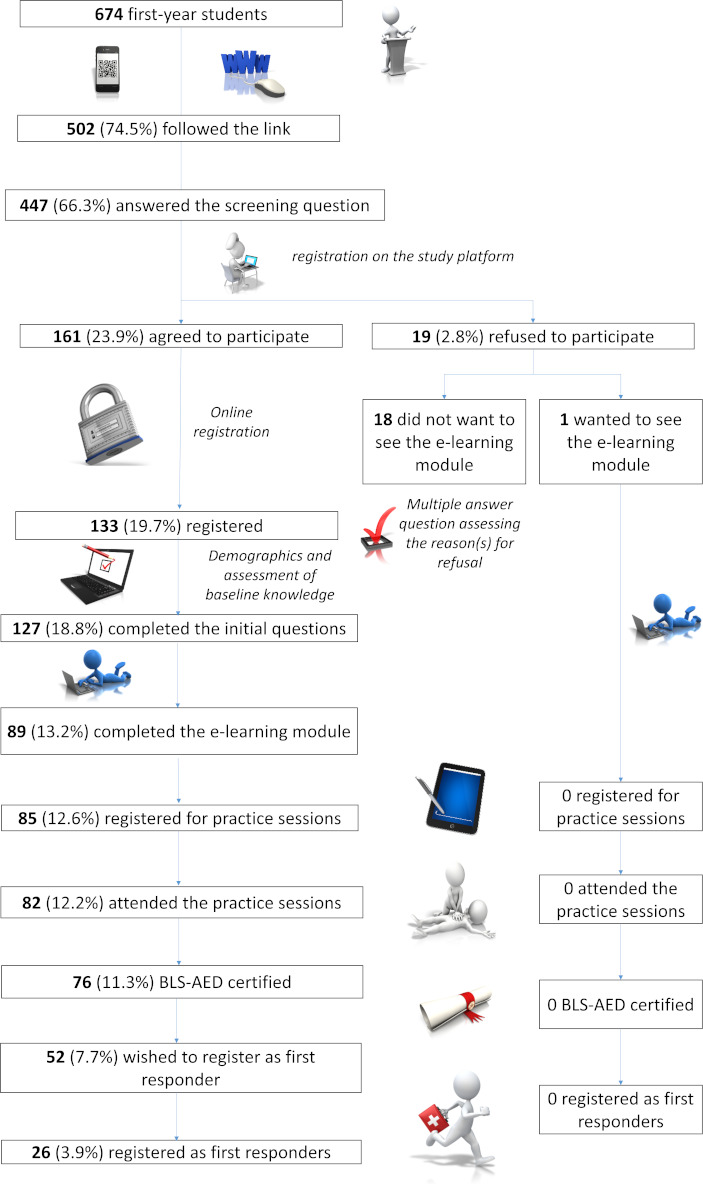
Study flowchart. AED: automatic external defibrillator; BLS: basic life support.

A total of 502 (74.5%) students followed the link directing them to the study platform, of whom 447 (66.3%) students completed the screening questionnaire. Only 133 (19.7%) students registered on the platform and 76 (11.3%) students received a BLS-AED certificate at the end of the learning program. In the postcourse questionnaire, 68.4% (52/76) of students who obtained the certificate indicated a desire to join the network of first responders, but only 34.2% (26/76) of students followed through. Figure 1 shows participation at each step of the study.

There was a statistically significant relationship between prior BLS knowledge and e-learning completion (*P*=.007), practical session attendance (*P*<.001), and obtention of a BLS certificate (*P*=.003). Conversely, there was no statistically significant relationship between prior BLS-AED knowledge and enrollment in the Save a Life network (*P*=.05) or interest in the program (*P*=.94).

Students’ confidence in their ability to initiate BLS maneuvers was significantly increased after following the learning path (*P*<.001, Multimedia Appendix 2). There was no statistical link between postcourse confidence and registration on the Save a Life platform (*P*=.09).

Postcourse satisfaction was 100% (76/76), as was the probability that students who had completed the learning path would recommend it to other students.

**Figure 2.**
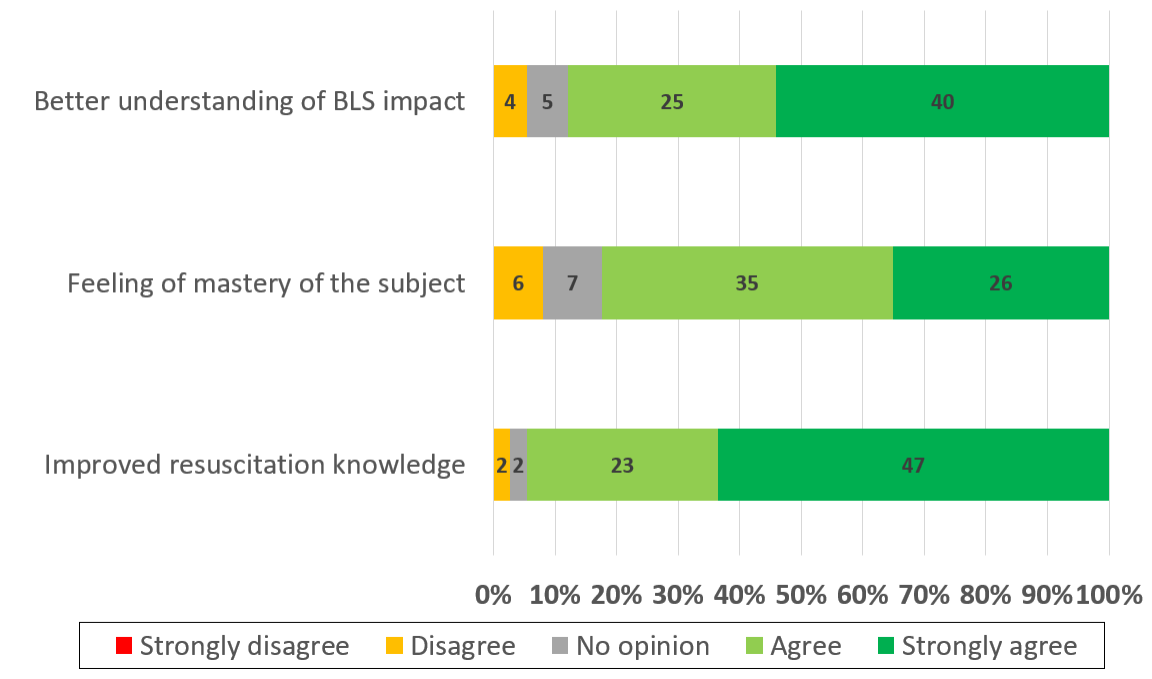
Factors promoting student confidence in their ability to perform basic life support (BLS) maneuvers.

Figure 2 shows that a better understanding of health issues, a feeling of mastery of the subject, and an improvement in knowledge regarding resuscitation all contributed to promoting participant confidence.

**Figure 3.**
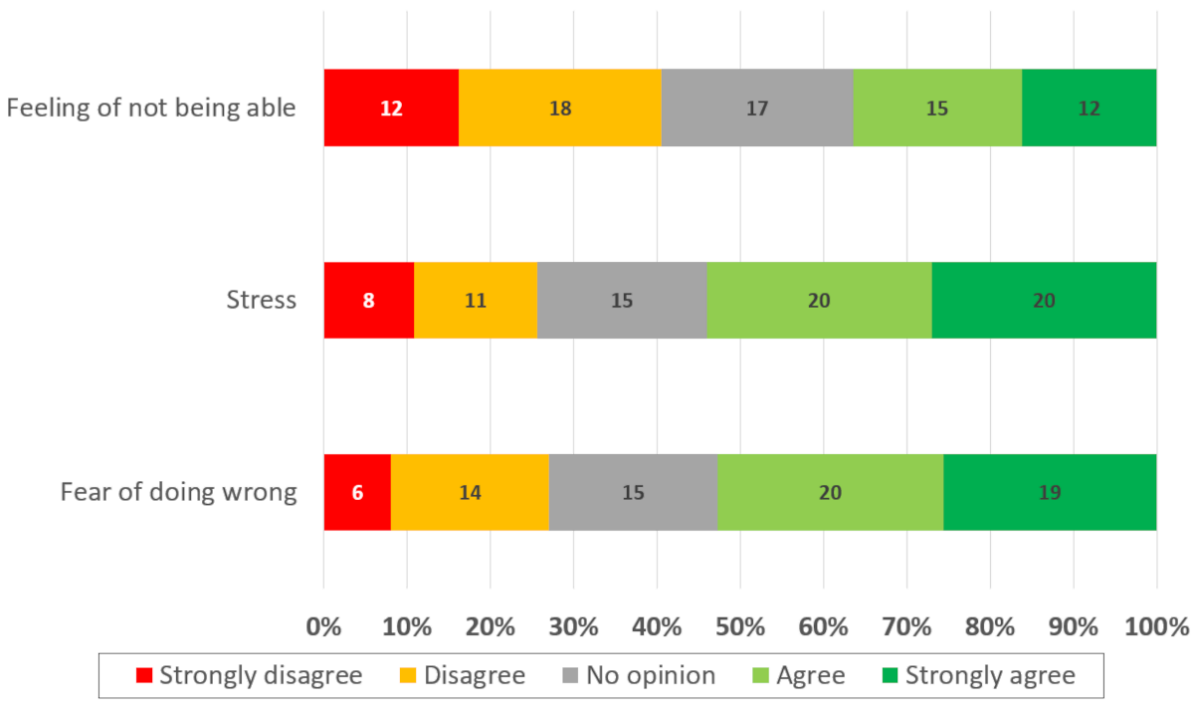
Factors limiting student confidence in performing basic life support maneuvers.

Stress and fear of doing wrong were the 2 main factors reported as limiting one’s confidence in performing BLS maneuvers (Figure 3).

**Figure 4.**
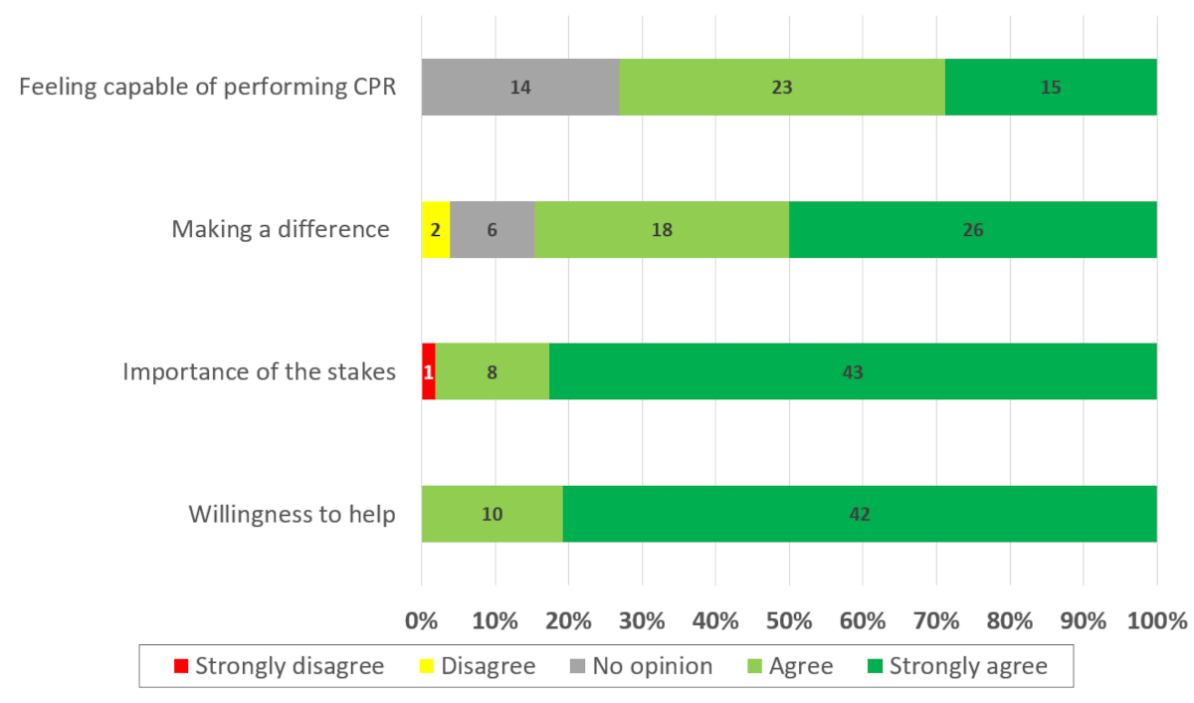
Factors promoting student willingness to register on the Save a Life platform. CPR: cardiopulmonary resuscitation.

Four factors promoting student willingness to register on the Save a Life platform were identified: feeling able to perform cardiopulmonary resuscitation, the possibility of making a difference, the stakes, and the desire to help (Figure 4).

**Figure 5.**
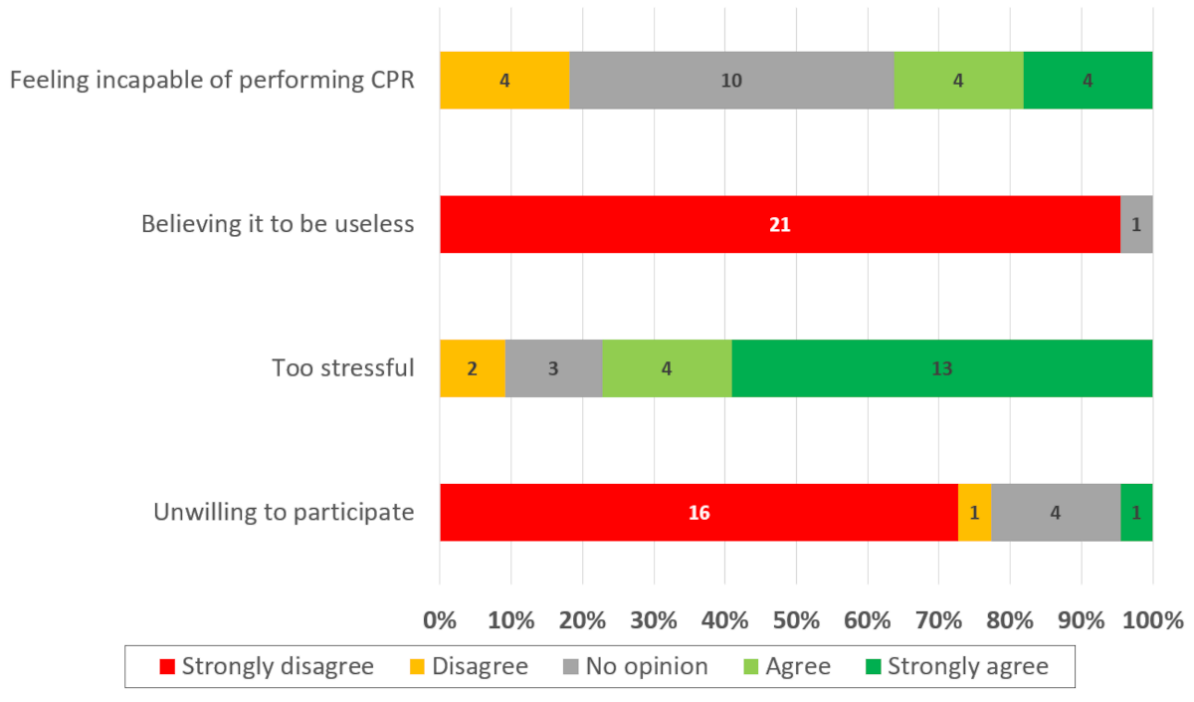
Factors limiting student willingness to register on the Save a Life platform. CPR: cardiopulmonary resuscitation.

Stress was the main factor preventing participants from registering as first responders (Figure 5).

## Discussion

### Main Considerations

Despite the modifications carried out according to the hypotheses outlined in the original study [16], and even after a second motivational intervention, recruitment was markedly lower than expected: indeed, in the original implementation study [16], the proportion of people who had registered on the platform at the end of the project was more than 2 times higher. The target set in the initial protocol was to recruit 10% of first-year students, a goal that has not yet been achieved.

These results deserve to be analyzed from a motivational point of view. The original study took place during the COVID-19 pandemic period, when student dynamics were probably different, and students may have been more inclined to participate in a presential activity, due to the fact that they had no choice but to spend their first year remotely. The COVID period was a major awareness and health involvement on the part of medical students. Their involvement in medical tasks having a perceptible impact on the future of patients affected by the pandemic undoubtedly positively influenced their motivational reinforcement. This paradox probably partially explains the low recruitment observed in this study, carried out outside the pandemic context. Other factors may also have influenced student motivation during the COVID pandemic: during this period, the health care system was highly regarded by the population, and the project may have given the students a sense of belonging [21]. The feeling of being useless in the face of what was happening and the desire to help may also have been stronger at this time [21]. Conversely, the end of the restrictions may have decreased their motivation to take part in such a project, and students may have been keen to resume many of the activities they had been deprived of [22].

The profile of the teacher endorsing the motivational intervention may also have played a role since teachers can have a significant influence on their students [23]. Since most medical students are interested in the clinical field, any advice, opinion, or encouragement given by a clinician could have a particularly important influence on students [24]. Clinicians can also share their interest and experience in a subject [25], and their support can foster student interest in a particular field [24]. Indeed, human beings strive to feel connected to those they admire, and the sense of belonging that a prestigious clinician radiates can influence student motivation [24]. In addition, first-year students are more motivated by success, prestige, and money, compared with the upper years, who are more focused on the personal gratification of their activity [24,26]. Moreover, student motivation fluctuates over the years, both qualitatively and quantitatively. Understanding its evolution can help encourage students to enjoy their learning and possibly improve their performance [27].

According to the theory of self-determination, there are several types of motivation, depending on what influences it and what goals it aims to achieve: intrinsic motivation, extrinsic motivation, and amotivation [26]. Intrinsic motivation is linked to personal interest in or pleasure inherent to the activity. Extrinsic motivation aims at a goal, a consequence separable from the subject, such as a reward or the absence of inconvenience [26]. Extrinsic motivation can be described as a continuum through which a process of internalization takes place, finally resulting in integrating action towards self-determination [26]. In the educational environment, motivation can be seen as having 3 determinants: the perception of the value of an activity, its skill, and its controllability [28].

A clinician’s valorization of the abilities and importance that each student can have in the health care system at their own level can influence the perception of their abilities, and their involvement and motivation [23]. A clinician’s speech on public health issues can have a greater impact and radiate a positive perception of the values involved [24,28].

Despite these different aspects motivating first-year students to participate in an optional learning program can still be difficult since it does not bring them any short-term benefits, in this case passing their exams. According to Dweck, students pursue learning goals as well as performance goals [29]. In the short term, when the risk of success is low, students would restrict themselves to the performance goal and neglect activities they consider ineffective for success [28,30].

A considerable proportion of students did not continue with the learning path after completing the questionnaire assessing their BLS knowledge. These students’ scores were lower than average, and their lack of knowledge may have had an impact on their perception of their skills for future activities, decreasing their self-confidence and motivation to continue their learning program [28]. This could be addressed by introducing BLS courses at school since, in Geneva, most schoolchildren receive only little, if any, first aid training before attending courses mandatory to obtain a driving license. Furthermore, training schoolchildren has been shown to improve OHCA outcomes [31]. Another option could be to remove this questionnaire from future studies to avoid any attrition linked to its administration.

Once enrolled in the learning program, students follow a certifying course, but registration on the Save a Life platform remains optional, and students therefore need further motivation to enlist as first responders. Participants agreed that perfecting their knowledge, mastering the subject, and understanding the health issues linked to early resuscitation all improved their self-confidence. This is in line with Viau and Louis’s [28] opinion, that is, that the perception of the value of an activity and of one’s own skills influence motivation.

Understanding the social impact of a first responder network could enable potential participants to internalize the values involved, and, according to the self-determination theory, increase motivation [26]. Thus, the societal impact of the Save a Life project could be further highlighted in future motivational interventions. This could improve recruitment since respondents unanimously agreed that the desire to help influenced their probability of registering as first responders.

Students reported that the main factors limiting their willingness to register as first responders were stress and the fear of making a mistake. Stress goes against the feeling of controllability of a situation, which is essential to self-confidence [28]. Addressing this issue will require further exploration, but a first step could be to point out the low risk of harm to the patient when practicing BLS maneuvers [14,15] and the clear benefits of early resuscitation early in the presentation [8-10].

Aspects modulating self-confidence need to be highlighted in future presentations to students, but also during the training program, to encourage these students as much as possible to join the Save a Life network. Their abilities and knowledge should be encouraged, and the efforts and gains they can make in the management of OHCA should be recognized. Any fears or doubts they may have must also be addressed during the learning path, and the effect of these motivational enhancements will need to be assessed in the next few years.

### Limitations

Since the very low participation rate could not be anticipated, the design of this study had to be adapted. Even though the second motivational intervention was endorsed by a clinician while the first was endorsed by a specialist in basic medical science, the effect of each specific intervention could not be assessed given the design of this study. A randomized controlled trial could be considered to explore the effects of endorsement by either type of specialist. In addition, the motivational interventions themselves were also different, and the effect of specifically designed and theory-based motivational interventions would also deserve to be determined. Finally, the impact of specific factors on motivation was only assessed among the students who had followed the learning path, thereby leading to a selection bias. Therefore, participatory research should be considered to help identify better recruitment strategies, and focus groups held to gather a more thorough and less biased understanding of students’ motivation and barriers to participation.

### Conclusions

Contrary to expectations, an earlier presentation during the academic year outside the COVID restriction period did not increase the recruitment of medical students as first responders, which was more than 2 times lower than in the implementation study even after further motivational interventions. A thorough quantitative and qualitative exploration of motivational factors should be carried out to determine potential ways of improving the recruitment of first-year medical students as first responders.

## Supplementary material

10.2196/63018 Multimedia Appendix 1 Web-based platform.

10.2196/63018 Multimedia Appendix 2 Evolution of self-confidence in practice basic life support maneuver.

## Abbreviations

AED: automatic external defibrillator
BLS: basic life support
OHCA: out-of-hospital cardiac arrest
UGFM: University of Geneva Faculty of Medicine
