# Supplementary material for: Recruiting Medical, Dental, and Biomedical Students as First Responders in the Immediate Aftermath of the COVID-19 Pandemic: Prospective Follow-Up Study
Source: JMIR Med Educ. 2025 Apr 24;11:e63018. doi: 10.2196/63018 (PMC12068746; doi:10.2196/63018)
Supplement: Multimedia Appendix 1 [file mededu-v11-e63018-s001.docx]

# Multimedia Appendix - Web-based platform

- The platform was developed using the Joomla 3.9 (Open Source Matters) content management system.
- Questionnaires were created using Community Surveys 5.9 (Shondalai, Bulasikku Technologies).
- Data was saved in an encrypted MySQL-compatible database (MariaDB 5.5.5, MariaDB Foundation) hosted on a Swiss server (KreativMedia GmbH)
