# Supplementary figures and images for: Recruiting Medical, Dental, and Biomedical Students as First Responders in the Immediate Aftermath of the COVID-19 Pandemic: Prospective Follow-Up Study
Source: JMIR Med Educ. 2025 Apr 24;11:e63018. doi: 10.2196/63018 (PMC12068746; doi:10.2196/63018)

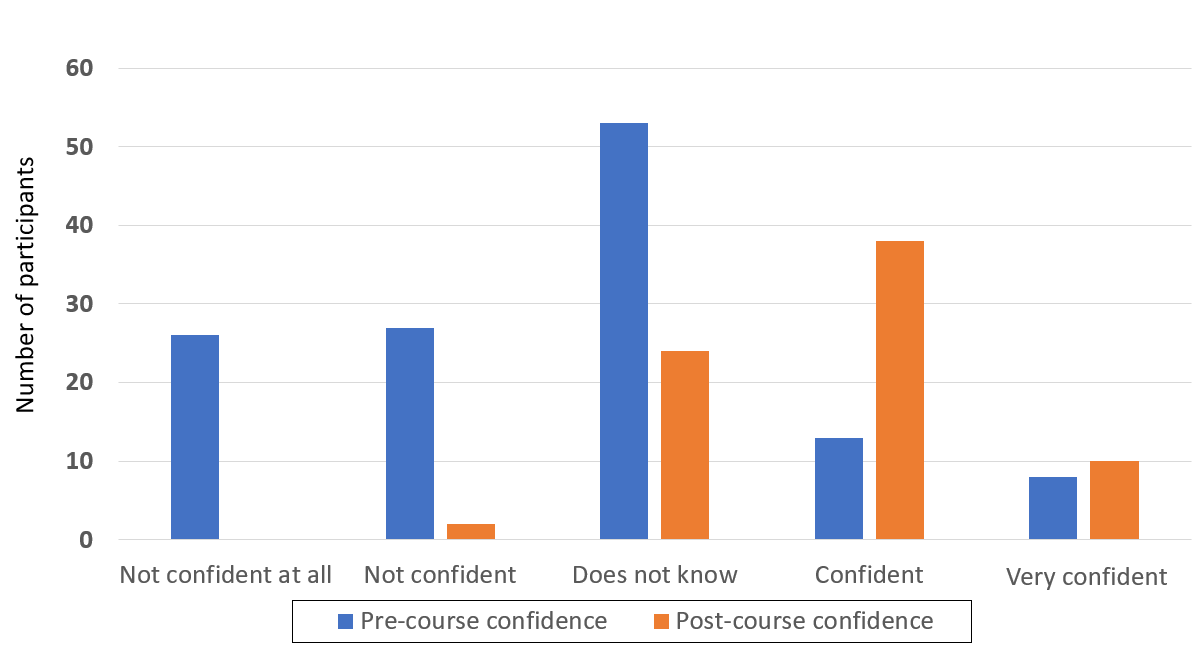

Supplement: Multimedia Appendix 2 [file mededu-v11-e63018-s002.png]
